# Centrilobular emphysema and coronary artery calcification: mediation analysis in the SPIROMICS cohort

**DOI:** 10.1186/s12931-018-0946-1

**Published:** 2018-12-18

**Authors:** Surya P. Bhatt, Hrudaya P. Nath, Young-il Kim, Rekha Ramachandran, Jubal R. Watts, Nina L. J. Terry, Sushil Sonavane, Swati P. Deshmane, Prescott G. Woodruff, Elizabeth C. Oelsner, Sandeep Bodduluri, MeiLan K. Han, Wassim W. Labaki, J. Michael Wells, Fernando J. Martinez, R. Graham Barr, Mark T. Dransfield, for the SPIROMICS investigators, Neil E. Alexis, Wayne H. Anderson, Eugene R. Bleecker, Richard C. Boucher, Russell P. Bowler, Elizabeth E. Carretta, Stephanie A. Christenson, Alejandro P. Comellas, Christopher B. Cooper, David J. Couper, Gerard J. Criner, Ronald G. Crystal, Jeffrey L. Curtis, Claire M. Doerschuk, Christine M. Freeman, Nadia N. Hansel, Annette T. Hastie, Eric A. Hoffman, Robert J. Kaner, Richard E. Kanner, Eric C. Kleerup, Jerry A. Krishnan, Lisa M. LaVange, Stephen C. Lazarus, Deborah A. Meyers, Wendy C. Moore, John D. Newell, Laura Paulin, Stephen Peters, Wanda K. O’Neal, Victor E. Ortega, Robert Paine, Nirupama Putcha, Stephen I. Rennard, Donald P. Tashkin, Mary Beth Scholand, J. Michael Wells, Robert A. Wise

**Affiliations:** 1 Division of Pulmonary, Allergy and Critical Care Medicine and Lung Health Center, University of Alabama at Birmingham, THT 422, 1720, 2nd Avenue South, Birmingham, AL 35294 USA; 2 UAB Lung Imaging Core, University of Alabama at Birmingham, Birmingham, AL 35294 USA; 3 Department of Radiology, University of Alabama at Birmingham, Birmingham, AL 35294 USA; 4 Department of Preventive Medicine, University of Alabama at Birmingham, Birmingham, AL 35294 USA; 5 Division of Pulmonary, Critical Care, Allergy and Sleep Medicine, University California San Francisco, San Francisco, CA 94143 USA; 6 Birmingham Veterans Affairs Hospital, Birmingham, AL 35294 USA; 7 Division of Pulmonary, Allergy and Critical Care Medicine, Columbia University Medical Center, New York, NY 10032 USA; 8 Division of Pulmonary and Critical Care Medicine, University of Michigan, Ann Arbor, MI 48109 USA; 9 Division of Pulmonary and Critical Care Medicine, Weill Cornell School of Medicine, New York, NY 10065 USA

**Keywords:** Emphysema, COPD, Coronary artery calcification, Cardiovascular disease, Mediators

## Abstract

**Background:**

Chronic obstructive pulmonary disease (COPD) is associated with a two-to-five fold increase in the risk of coronary artery disease independent of shared risk factors. This association is hypothesized to be mediated by systemic inflammation but this link has not been established.

**Methods:**

We included 300 participants enrolled in the SPIROMICS cohort, 75 each of lifetime non-smokers, smokers without airflow obstruction, mild-moderate COPD, and severe-very severe COPD. We quantified emphysema and airway disease on computed tomography, characterized visual emphysema subtypes (centrilobular and paraseptal) and airway disease, and used the Weston visual score to quantify coronary artery calcification (CAC). We used the Sobel test to determine whether markers of systemic inflammation mediated a link between spirometric and radiographic features of COPD and CAC.

**Results:**

FEV_1_/FVC but not quantitative emphysema or airway wall thickening was associated with CAC (*p* = 0.036), after adjustment for demographics, diabetes mellitus, hypertension, statin use, and CT scanner type. To explain this discordance, we examined visual subtypes of emphysema and airway disease, and found that centrilobular emphysema but not paraseptal emphysema or bronchial thickening was independently associated with CAC (*p* = 0.019). MMP3, VCAM1, CXCL5 and CXCL9 mediated 8, 8, 7 and 16% of the association between FEV_1_/FVC and CAC, respectively. Similar biomarkers partially mediated the association between centrilobular emphysema and CAC.

**Conclusions:**

The association between airflow obstruction and coronary calcification is driven primarily by the centrilobular subtype of emphysema, and is linked through bioactive molecules implicated in the pathogenesis of atherosclerosis.

**Trial Registration:**

ClinicalTrials.gov: Identifier: NCT01969344.

**Electronic supplementary material:**

The online version of this article (10.1186/s12931-018-0946-1) contains supplementary material, which is available to authorized users.

## Introduction

Chronic obstructive pulmonary disease (COPD) is associated with a two-to-five fold increase in the risk of coronary artery disease [1, 2] and epidemiologic studies indicate that this relationship is independent of shared risk factors such as age and cigarette smoking [1, 2]. The magnitude of risk of cardiac disease associated with low lung function is similar to the population-attributable risk conferred by well-established cardiac risk factors such as hypertension and diabetes mellitus, suggesting that COPD is a proatherogenic condition [3]. Multiple studies have shown associations between low lung function and CT emphysema with measures of endothelial dysfunction and atherosclerosis including brachial flow-mediated dilation, [4] carotid intima-medial thickness, [5] arterial stiffness, [6, 7] and coronary artery calcium (CAC) [8, 9] and though some have also suggested a dose-response relationship [10, 11] the underlying mechanistic links are not clear.

The most accepted hypothesis for the link between COPD and atherosclerosis is that chronic inflammation in the lungs results in systemic inflammation and contributes to an atherogenic milieu [12]. The systemic inflammation observed in other chronic diseases is associated with atherosclerosis, [13] and it is plausible that it is the prime driver of coronary artery disease in COPD. However, studies examining the link between poor lung function and systemic inflammation have demonstrated conflicting results or weak to modest correlations [6, 7, 10, 14–16]. No single study has assessed a three-way link between airflow obstruction, systemic inflammation and coronary artery disease in COPD, and no specific mediators of these associations have been identified. COPD includes both emphysema and airway disease, and it is not known if these structural phenotypes are differentially associated with systemic inflammation and atherosclerosis, and whether this underlies the poor correlations reported.

We analyzed data from the Subpopulations and intermediate outcome measures in COPD study (SPIROMICS) and hypothesized that the severity of airflow obstruction would be directly associated with coronary artery disease as assessed by CAC. We also hypothesized that emphysema subtypes and airway disease are differentially associated with markers of systemic inflammation which would mediate the link with coronary artery disease.

## Methods

### Participants

We included participants from SPIROMICS, a well characterized cohort of normal subjects (stratum 1), current and former smokers at risk for COPD (stratum 2), with mild-to-moderate COPD as defined by the Global Initiative for Chronic Obstructive Lung Disease (GOLD) recommendations (stratum 3), and with severe-to-very-severe COPD (stratum 4) [17]. The study was approved by the institutional review boards of all eleven participating centers, and all participants provided written informed consent. The first 75 participants who enrolled in each stratum with a full set of biomarker data were included. All participants underwent pre and post bronchodilator spirometry at enrollment according to the American Thoracic Society guidelines. Computed tomography (CT) scans were performed at end inspiration. Physician diagnosed self-reported hypertension, diabetes mellitus, and coronary artery disease (CAD) were recorded. Statin use was defined by patient-reported use of statins in the 3 months prior to enrollment. We used CAC as a surrogate for clinical and subclinical CAD. Compared to cardiovascular risk scores, CAC is a robust measure of early and subclinical CAD and is also an independent predictor of incident adverse cardiovascular events [18].

### Measurement of lung disease

Airflow obstruction was quantified by the ratio of the post bronchodilator forced expiratory volume in the first second (FEV_1_) to the forced vital capacity (FVC), and the severity of airflow obstruction quantified by FEV_1_%predicted [19]. We measured overall emphysema quantitatively using 3D Slicer software (https://www.slicer.org/) on inspiratory CT with a density mask analysis after segmentation and exclusion of large and medium sized airways, such that voxels <− 950 HU in density were classified as emphysematous [20]. Airway wall thickness was quantified by the Pi10, defined by the square root of the wall area of a theoretical circular cross section of an airway with 10 mm lumenal perimeter, using an automated algorithm based on the using the optimal surface algorithm (Apollo Software, Vida Diagnostics Inc) [21, 22]. Five readers (4 radiologists and 1 pulmonologist) visually scored emphysema subtypes and airway disease on lung windows according to the Fleischner Society criteria [23]. Briefly, emphysema subtypes were classified as centrilobular emphysema or CLE (none, trace, mild, moderate, confluent and advanced destructive) and paraseptal emphysema or PSE (none, mild, substantial). The presence of any moderate, confluent or advanced destructive CLE was deemed clinically substantial. The presence or absence of airway wall thickening was noted and when present, severity classified as borderline or definite. Definite airway wall thickening was considered clinically significant.

### Measurement of potential plasma mediators

Ten plasma mediators of the link between COPD and CAC were selected a priori representing pathways previously implicated as potential drivers of either disease. Based on plausible biologic pathways, the mediators included were acute phase reactants and cytokines involved in systemic inflammation [C-reactive protein (CRP), fibrinogen, tumor necrosis factor-alpha (TNF-α) and interleukin-6 (IL-6)], chemokines involved in neutrophil activation [C-X-C Motif Chemokine Ligand 5 (CXCL5)], T-cell chemoattractant [C-X-C Motif Chemokine Ligand 9 (CXCL9)], adhesion molecules important in the leucocyte-endothelial interaction [intercellular (ICAM-1) and vascular cell (VCAM-1) adhesion molecules], and matrix metalloproteinases important in emphysema and coronary plaque pathogenesis [MMP3 and MMP9]. Although we had access to a number of additional plasma biomarkers, we did not include these in the analysis to avoid multiple comparisons and false discovery. All plasma mediators were measured using commercially available Myriad RBM “Human InflammationMAP” multiplex assay [24].

### Measurement of coronary artery calcification

High resolution CT scans were performed in all participants and inspiratory scans were used for the visual measurement of CAC. Two readers who were blinded to clinical and quantitative CT metrics visually analyzed the coronary arteries using mediastinal soft tissue window settings to calculate the Weston visual score for CAC [25]. The Weston score ranges from 0 to 3 for each of the left main, left circumflex, anterior descending and the right coronary arteries as follows: No visually detected calcium = 0, Only a single high density pixel detected = 1, Calcium dense enough to cause a blooming artifact =3, and Calcium intermediate and between 1 and 3 = 2. The total score ranges from 0 to 12. The Weston score on non-gated scans correlates well with Agatston scores measured on electrocardiographically gated CT scans [26]. We excluded participants who had undergone coronary artery bypass grafting.

### Statistical analyses

Distributions of continuous variables were tested and log transformations were performed for emphysema, CAC and plasma mediators. Pearson’s correlation was used to test correlations between FEV_1_/FVC, Pi10, log emphysema, the log of plasma mediators and log CAC. A sample size of 300 patients was considered adequate to achieve 88% power to detect indirect effect size of 0.04 or larger when direct effect is 0.1 and total effect is 0.14 at significance level of alpha = 0.05. The power of the test of the indirect effect was prepared by using the joint test of significance [27]. Intra- and inter-rater agreement were calculated for continuous and categorical CT parameters using intra-class correlation coefficient and multi-rater kappa, respectively. Univariate and multivariable associations between airflow obstruction (post bronchodilator FEV_1_/FVC) and log CAC were tested with adjustment for age, gender, race, body-mass-index (BMI), pack-years of smoking, current smoking status, diabetes mellitus, hypertension, statin use, and CT scanner type using generalized linear regression models. Similarly, multivariable associations between quantitative log emphysema and log CAC were tested with adjustment for age, gender, race, body-mass-index (BMI), postbronchodilator FEV_1_, pack-years of smoking, current smoking status, diabetes mellitus, hypertension, statin use, and CT scanner type. Similar calculations were repeated for Pi10, emphysema subtypes and the presence of bronchial wall thickening in separate models.

#### Mediation analysis

To test whether any of the preselected plasma biomarkers were important in the link between emphysema and CAC, we performed mediation analysis by using three way regression models. The Sobel test was used to determine whether any specific biomarker fully or partially mediated the relationship between FEV_1_/FVC and log CAC (Additional file 1: Figure S1). The following criteria had to be met for three way regression models for mediation analysis: there existed a significant association between the independent and dependent variable; significant association between the independent variable and the mediator; significant association between the mediator and the dependent variable; and the association between the independent and dependent variables is attenuated when the mediator is added to the regression model. The indirect and direct effects of FEV_1_/FVC on log CAC were evaluated based on correlation coefficients of FEV_1_/FVC estimated from three way regression models. Sobel test was used to determine if the indirect effect of FEV_1_/FVC was statistically significant. Mediated proportions of the total effect of FEV_1_/FVC were calculated and presented. As the Sobel test assumes normality of variables and that there are no measurement errors, percentile bootstrap confidence intervals were used to test the indirect effect. The accelerated bias-corrected bootstrap estimates correction for a bias in the average estimate and the standard deviation across potential values of the indirect coefficient [28]. Similar analyses were repeated for the association between structural lung disease significantly associated with log CAC on multivariable analysis (centrilobular emphysema) and log CAC.

All analyses were performed using IBM SPSS Statistics 24.0 and SAS 9.4 (SAS Institute, Cary, NC). A two-sided alpha level of 0.05 was considered statistically significant for all analyses.

## Results

We included 300 participants, 75 from each stratum with a full set of biomarkers and complete spirometry and CT data. The mean (SD) age was 62.0 (9.6) years, with 36.7 (28.3) pack-years smoking history. 209 (69.7%) were male, 91 (30.3%) were African American, and 94 (31.3%) were current smokers. Kappa values for intra-observer agreement for detecting substantial CLE, substantial PSE, and definite airway wall thickening were 0.88 ± SE0.09, 0.54 ± 0.23, and 0.53 ± 0.24, respectively, and for inter-observer agreement were 0.81 ± 0.08, 0.37 ± 0.08, and 0.33 ± 0.08, respectively. There was excellent agreement between readers for CAC, with intra-class correlation coefficient for within and between observers of 0.98 (95% CI 0.97 to 0.98; *p* < 0.001), and 0.96 (95% CI 0.92 to 0.98; *p* < 0.001), respectively. Table 1 shows a comparison of baseline characteristics of these participants. Participants with COPD had a greater frequency of CAD than those without COPD (11.3% vs. 2.0%; *p* = 0.002), and those with COPD had a greater CAC score than those without COPD (median 6.0, IQR_25–75_ 3.0 to 10.0 vs. 2.0, IQR_25–75_ 0 to 5.0; *p* < 0.001). 137 (45.7%) had hypertension, 43 (14.3%) had diabetes mellitus, and 121 (40.3%) were on statins. With progressive airflow obstruction across strata, there was greater emphysema as expected, as well as increasing CAC (Jonckheere’s trend test *p* < 0.001).

**Table 1:** Comparison of Demographics and Computed Tomography Characteristics of Participants

	Stratum 1 (*n* = 75)	Stratum 2 (*n* = 75)	Stratum 3 (*n* = 75)	Stratum 4 (*n* = 75)
Age (years)	54.3 (9.5)	59.8 (9.3)	64.8 (8.5)	64.8 (6.7)
Sex, Male (%)	32 (42.7%)	56 (74.7%)	64 (85.3%)	57 (76.0%)
Race, White (%)	49 (65.3%)	50 (66.7%)	66 (88.0%)	63 (84.0%)
BMI (kg/m^2^)	28.4 (5.5)	28.3 (4.6)	27.8 (4.0)	26.6 (4.8)
Smoking Pack-years	0 (0)	40.6 (17.5)	49.8 (22.4)	56.5 (21.9)
Hypertension (%)	24 (32%)	31 (41.3%)	42 (56%)	40 (53.3%)
Diabetes mellitus (%)	4 (5.3%)	10 (13.3%)	19 (25.3%)	10 (13.3%)
FEV_1_ (L)	3.06 (0.69)	3.24 (0.71)	2.79 (0.62)	1.23 (0.26)
FEV_1_% pred	102.2 (12.1)	101.1 (11.2)	87.0 (15.8)	39.8 (8.1)
FVC (L)	3.76 (0.90)	4.18 (0.92)	4.46 (0.90)	3.20 (0.76)
FVC %pred	97.8 (11.1)	99.9 (11.1)	104.9 (15.6)	77.5 (14.4)
FEV_1_/FVC	0.82 (0.05)	0.78 (0.05)	0.62 (0.06)	0.39 (0.09)
Percentage Emphysema	1.6 (1.7)	2.0 (1.8)	5.2 (5.1)	17.6 (11.4)
Pi10	3.67 (0.09)	3.68 (0.07)	3.70 (0.08)	3.74 (0.08)
CAC	2.0 (2.8)	3.6 (3.5)	6.5 (4.2)	6.5 (4.0)
Statins^a^ (%)	19 (25.3%)	25 (33.3%)	45 (60.0%)	32 (42.7%)
Centrilobular Emphysema (%)				
Mild (yes/no) (%)	3 (4%)	13 (17.3%)	20 (26.7%)	8 (10.7%)
Moderate (yes/no) (%)	0 (0%)	4 (5.3%)	21 (28%)	22 (29.3%)
Advanced/Confluent (yes/no) (%)	0 (0%)	4 (5.3%)	17 (22.7%)	42 (56%)
Substantial Paraseptal Emphysema (yes/no) (%)	0 (0%)	10 (13.3.%)	16 (21.3%)	13 (17.3%)
Definite Bronchial Wall Thickening (yes/no) (%)	0 (0%)	5 (6.7%)	17 (22.7%)	29 (38.7%)

All values shown as mean (standard deviation) unless otherwise stated

*FEV*_*1*_ Forced expiratory volume in the first second, *FVC* Forced vital capacity, *CAC* Coronary artery calcification, *Pi10* Square root of the wall area of a theoretical circular cross section of an airway with 10 mm lumenal perimeter

Stratum 1 = Lifetime non-smokers

Stratum 2 = Smokers without airflow obstruction. FEV_1_/FVC ≥0.70

Stratum 3 = Mild to moderate airflow obstruction. FEV_1_/FVC < 0.70, FEV_1_ ≥ 50%predicted

Stratum 4 = Severe to very severe airflow obstruction. FEV_1_/FVC < 0.70, FEV_1_ < 50%predicted

^a^Medication data displayed for usage in the three months prior to enrollment

### Spirometric airflow obstruction

There was a significant association between airflow obstruction (FEV_1_/FVC) and log CAC after multivariable adjustments (Table 2). On univariate analysis, the severity of airflow obstruction (FEV_1_%predicted) was also associated with log CAC (crude β = − 0.004 ± 0.001; *p* < 0.001), and this relationship remained significant after multivariable adjustment (adjusted β = − 0.001 ± 0.001; *p* = 0.009).

**Table 2:** Associations of Chronic Obstructive Pulmonary Disease with Coronary Artery Calcium^a^

	Univariate		Multivariable^b^	
	β estimate ±SE	*p* value	β estimate ±SE	*p* value
FEV_1_/FVC	−0.723 ± 0.006	< 0.001	−0.192 ± 0.093	0.039
Log Emphysema^c^	0.348 ± 0.064	< 0.001	−0.075 ± 0.076	0.326
Pi10	0.841 ± 0.207	< 0.001	0.049 ± 0.214	0.819
Centrilobular Emphysema	0.245 ± 0.034	< 0.001	0.073 ± 0.036	0.042
Paraseptal Emphysema	0.102 ± 0.052	0.050	0.031 ± 0.039	0.429
Airway Wall Thickening	0.195 ± 0.046	< 0.001	0.059 ± 0.037	0.112

*FEV*_*1*_ Forced expiratory volume in the first second, *FVC* Forced vital capacity. *Pi10* Square root of the wall area of a theoretical circular cross section of an airway with 10 mm lumenal perimeter

^a^Coronary artery calcification log transformed. Separate models run for association between CAC and each subtype of chronic obstructive pulmonary disease

^b^Model adjusted for age, sex, race, BMI, smoking status, pack-years, FEV_1_, hypertension, diabetes mellitus, statin use, and CT scanner type, except model for FEV_1_/FVC where FEV_1_ was not included

^c^Assessed by percentage of low attenuation areas <-950HU on quantitative CT using density mask analysis

### Quantitative emphysema and airway disease

On univariate analysis, there was a significant association between log emphysema and log CAC (crude regression coefficient β = 0.348 ± 0.064; *p* < 0.001). However, after adjustment for age, gender, race, BMI, pack-years of smoking, current smoking status, FEV_1_, diabetes mellitus, hypertension, statin use, and CT scanner type, this association was no longer significant, adjusted regression coefficient β = − 0.074 ± 0.077; *p* = 0.340 (Table 2). Pi10 was significantly associated with log CAC on univariate analysis, but this association was no longer significant after multivariable adjustments (Table 2).

### Visual analysis of COPD subtypes

We found a significant association between log CAC and substantial CLE (adjusted β = 0.084 ± 0.036; *p* = 0.019) but not paraseptal emphysema on multivariable analysis (Table 2). Although there was a significant association between definite airway wall thickening and log CAC on univariate association, this association was no longer significant after multivariable adjustment (Table 2).

### Plasma biomarkers

Additional file 1: Table S1 shows the mean (SD) values for the ten preselected biomarkers across the range of normal and disease severity. Most of the selected biomarkers increased with severity of lung disease, except CXCL5 which was lower in those with more severe airflow obstruction. Additional file 1: Table S2 shows that multiple plasma mediators correlated significantly with both CAC and emphysema (CRP, fibrinogen, MMP3, CXCL9 and VCAM1). CXCL5 correlated inversely with both CAC and emphysema. Additional file 1: Table S3 shows comparisons of biomarkers between those with and without each visual subtype of COPD. Multiple biomarkers were substantially higher in those with substantial CLE than in those without CLE, whereas there were no differences in biomarkers by substantial PSE status.

### Mediation analysis

Table 3 shows the results of mediation analyses. We found a partial mediation effect for MMP3, VCAM1, CXCL5 and CXCL9 for the association between FEV_1_/FVC and log CAC, and these biomarkers partially mediated approximately 8, 8, 7 and 16% of the effect, respectively. As log CAC was associated with substantial centrilobular emphysema, but not airway wall thickening, paraseptal emphysema, or overall quantitative emphysema, we repeated mediation analysis for the link between substantial CLE and log CAC. We found a partial mediation effect for three of the same plasma biomarkers important in the association between FEV_1_/FVC and log CAC: MMP3, VCAM1 and CXCL9, for the association between substantial CLE and log CAC. Approximately 8, 7 and 17% of the total effect of substantial CLE on log CAC was partially mediated by MMP3, VCAM1 and CXCL9, respectively. As the association between airway wall thickening and log CAC approached statistical significance, we repeated mediation models for this association. None of the plasma biomarkers mediated this association.

**Table 3:** Plasma Mediators of the Association between Chronic Obstructive Pulmonary Disease and Coronary Artery Calcification

Mediator^a^	Indirect Effect	Bootstrap Bias Corrected 95% CI for Indirect Effect	Direct Effect	% Effect Mediated	Sobel *p*-value
A. Airflow obstruction (FEV_1_/FVC) and CAC					
MMP3 (ng/mL)	−0.061	−0.126 to − 0.022	−0.662	8.5	0.015
VCAM1 (ng/mL)	−0.057	−0.134 to − 0.020	−0.666	7.9	0.019
CXCL9 (pg/mL)	−0.114	−0.194 to − 0.057	−0.609	15.8	< 0.001
CXCL5 (ng/mL)	−0.049	−0.102 to − 0.015	−0.673	6.8	0.040
B. Centrilobular Emphysema and CAC					
MMP3 (ng/mL)	0.019	0.006 to 0.042	0.226	7.9	0.033
VCAM1 (ng/mL)	0.018	0.004 to 0.041	0.227	7.3	0.041
CXCL9 (pg/mL)	0.042	0.022 to 0.074	0.203	17.0	0.001

*CAC* Coronary artery calcification, *FEV*_*1*_ Forced expiratory volume in the first second, *FVC* Forced vital capacity, *MMP3* Matrix metalloproteinase 3. VCAM1 = Vascular cell adhesion molecule 1. CXCL = C-X-C Motif Chemokine Ligand

^a^All mediators were log transformed prior to analysis

## Discussion

In a cohort of healthy non-smokers and COPD patients with a range of disease severity, we found a significant relationship between airflow obstruction and the severity of coronary artery calcification but no association with structural correlates on quantitative CT imaging. The association between airflow obstruction and coronary calcification appears to be driven primarily by the centrilobular subtype of emphysema, and is linked through the same mediators, common bioactive molecules implicated in the pathogenesis of atherosclerosis.

There is growing awareness that COPD is a pro-atherosclerotic condition with an attributable risk comparable to other chronic inflammatory conditions such as rheumatoid arthritis, as well as other traditional cardiac risk factors such as diabetes mellitus and hypertension [1, 2, 29]. We found that airflow obstruction is associated with the severity of CAC, a finding that supports previous epidemiologic studies showing a higher frequency of coronary artery disease and greater burden of coronary calcification in COPD [8, 9, 30, 31]. Although there appears to be a dose-response relationship between airflow obstruction and cardiac disease, previous studies such as the Evaluation of COPD Longitudinally to Identify Predictive Surrogate End-points (ECLIPSE) and the Multi-Ethnic Study of Atherosclerosis (MESA) have not found a relationship between the structural correlates of COPD such as emphysema and coronary calcification [32, 33]. We found a similar discordance with a relationship between airflow obstruction and CAC, but not between quantitative measures of structural lung disease and CAC. This has been a perplexing finding given the known relationship between airflow obstruction and CAC. There is however significant disagreement between quantitative emphysema and visual emphysema subtypes and visual subtypes offer prognostic additional information over quantitative measures [34]. We extend the literature by reporting that although overall emphysema and airway disease are not associated with CAC, the centrilobular subtype of emphysema, but not paraseptal emphysema or airway wall thickness, is independently associated with CAC.

The mediators of the link between airflow obstruction and coronary artery disease are not clear, and systemic inflammation has been commonly implicated in the pathogenesis of cardiac disease, mostly based on data from other chronic inflammatory conditions [13]. The correlations reported between inflammatory markers and measures of atherosclerosis in COPD are either modest or non-significant [6, 7, 15, 32, 35–37]. These previous studies were not designed to examine three-way relationships between COPD, cardiac disease and systemic inflammation. We identified a number of biomarkers that mediate the link between airflow obstruction and CAC, and found that the mediators of the link between centrilobular emphysema and CAC were mostly similar. Similar to previous studies showing weak links between CAC and non-specific markers of inflammation such as CRP and fibrinogen in COPD, we did not find any mediator effect for these biomarkers. Matrix metalloproteinases are implicated in the pathogenesis of both emphysema and coronary atherosclerosis. MMP3 is present and enzymatically active in coronary plaques, and MMP3 polymorphisms are associated with the initiation and progression of these plaques, with plaque instability as well as with coronary calcification [38–40]. Matrix metalloproteinases 1 and 9 are important in the stability of the extracellular matrix in various tissues including the lung; however the role of MMP3 in the pathogenesis of emphysema is less clear. Recent studies have suggested that MMP3 polymorphisms are also important in the pathogenesis and severity of emphysema [41, 42]. Soluble adhesion molecules are involved in recruitment of macrophages and neutrophils, are involved in the transendothelial migration of these inflammatory cells, and circulating levels are higher in COPD patients than in healthy non-smokers [43]. VCAM1 but not ICAM1 has been implicated in the early pathogenesis of atherosclerotic plaques, via promotion of monocyte adhesion and accumulation on vessel walls susceptible to developing atherosclerosis [44]. Circulating VCAM is associated with greater CAD risk in other chronic inflammatory conditions such as type 2 diabetes mellitus [45, 46]. CXCL9, a chemokine, is a T-cell chemoattractant that is induced by IFN-γ, and levels of CXCL9 are greater in sputum of COPD patients, and these levels correlate with disease severity [47]. CXCL9 and other CXCR3 chemokines have been implicated in the regulation of migration of monocytes and lymphocytes and their retention in atherosclerotic lesions, and serum levels of CXCL9 correlate with severity of coronary narrowing as well as coronary artery calcification [48, 49]. Although mediation analysis cannot establish causality, and it is possible that systemic elevation of these biomarkers may just reflect progressive atherosclerosis, results of the mediation analyses suggest that they may have bioactive properties that are important in the pathogenesis of cardiovascular disease specifically in COPD. CXCL5 decreased with worsening severity of airflow obstruction. Recent data show that contrary to the other chemokines, CXCL5 may facilitate cholesterol efflux from macrophages and by reducing macrophage foam cell formation, have a protective role in atherogenesis [50].

Our study has several strengths. Participants in this cohort are well characterized with extensive phenotyping using CT and spirometry and a wide array of biomarkers. We included normal controls as well as participants with a wide range of airflow obstruction. The study also has some limitations. Although we selected ten potential mediators based on biologic plausibility and prior evidence, we may have missed other potential mediators; however, we did not include all available biomarkers so as to not overfit the models and to avoid false positive discovery. The inter-rater agreement for centrilobular emphysema was excellent and fair for paraseptal emphysema and bronchial wall thickening; however, we also used Pi10 to quantify airway disease. These rates of agreement for bronchial wall thickening are comparable to those observed in previous studies [51, 52]. Our associations are cross-sectional, and although temporal associations with acute events are an alternate model, these models would miss a substantial number of subclinical disease. Information on comorbidities such as diabetes mellitus and hypertension were ascertained from the participants as self-reported physician diagnoses but not confirmed with medical records. Although we selected participants randomly, there was a preponderance of male participants in the study and this may limit the generalizability of our findings. Finally, although mediation analyses were performed, we do not attribute causality and more research is needed to confirm these biologic pathways.

## Conclusions

Chronic obstructive pulmonary disease is associated with coronary artery calcification, and the association is mainly driven by the centrilobular emphysema subtype. We found that inflammatory biomarkers involved in coronary atherosclerosis are elevated in those with COPD, especially with centrilobular emphysema, and mediate the link between emphysema and coronary artery calcification.

## Additional file

Additional file 1: Supplemental Information. (DOCX 73 kb)

## Abbreviations

BMI: Body mass index
CAC: Coronary artery calcification
CAD: Coronary artery disease
CLE: Centrilobular emphysema
COPD: Chronic obstructive pulmonary disease
CRP: C-reactive protein (CRP)
CT: Computed tomography
CXCL: C-X-C Motif Chemokine Ligand
FEV1: Forced expiratory volume in the first second
FVC: Forced vital capacity
HU: Hounsfield Unit
ICAM: Intercellular adhesion molecule
IL-6: Interleukin-6
MMP: Matrix metalloproteinase
Pi10: Square root of the wall area of a theoretical circular cross section of an airway with 10 mm lumenal perimeter
PSE: Paraseptal emphysema
TNF-α: Tumor necrosis factor-alpha
VCAM: Vascular cell adhesion molecule
