# Supplementary material for: Centrilobular emphysema and coronary artery calcification: mediation analysis in the SPIROMICS cohort
Source: Respir Res. 2018 Dec 18;19:257. doi: 10.1186/s12931-018-0946-1 (PMC6299495; doi:10.1186/s12931-018-0946-1)
Supplement: Supplementary file 1 — Supplemental Information. (DOCX 73 kb) [file 12931_2018_946_MOESM1_ESM.docx]

**Methods**

**CT Protocol:**

CT data was acquired at 6 centers using multiple scanners. To ensure comparability, SPIROMICS adapted a standardized protocol which has been previously described.^1^ Briefly, scanners included Siemens (Definition and Sensation), General Electric (Discovery) and Phillips (Brilliance). The imaging protocol consisted of slice thickness of 0.625 to 0.75 mm, slice spacing of 0.5 mm, and pitch of 0.923 to 1.0. Exposure parameters were 120 kV and rotation of 0.5 seconds, and 80-270 mAs for inspiratory scans and 50-145 mAs for expiratory scans, depending on BMI. Reconstruction kernels included Philips B, GE Standard, and Siemens B35 kernels. The Genetic Epidemiology of COPD (COPDGene) phantom test object was scanned at regular intervals to ensure calibration of CT scanners. CT scanners have remained within 4 Hounsfield Units (HU) for internal air measurements, and within 3 HU for external air measurements.

Images were analyzed using the Apollo software (VIDA Diagnostics, Coralville, IA).^2^ Percentage emphysema was calculated using the percentage of lung or lobe volume at TLC with attenuation less than -950 Hounsfield Units (HU). Airway disease was quantified using the Pi10, the square root of the wall of area of a theoretical airway with internal perimeter of 10 mm.^3,4^ The Pi10 measurements were made in an automated fashion based on airway measurements performed with the optimal surface algorithm. This algorithm does not suffer from overestimated wall thicknesses in small airways compared with full-width half-maximum.^5,6^

**Additional file 1: Table S1: Comparison of Plasma Biomarkers**

|  | **Stratum 1**  **(n=75)** | **Stratum 2**  **(n=75)** | **Stratum 3**  **(n=75)** | **Stratum 4**  **(n=75)** |
| --- | --- | --- | --- | --- |
| **CRP****  **(ug/mL)** | 3.3 (6.0) | 4.1 (6.5) | 4.1 (3.9) | 7.2 (9.9) |
| **Fibrinogen****  **(mg/mL)** | 4.4 (0.9) | 4.7 (1.1) | 4.9 (1.2) | 5.2 (1.3) |
| **IL-6****  **(pg/mL)** | 3.8 (1.7) | 5.3 (13.6) | 8.7 (32.0) | 23.6 (164.6) |
| **TNF-α**  **(pg/mL)** | 9.1 (1.4) | 9.2 (1.3) | 10.0 (3.9) | 9.5 (3.2) |
| **MMP3****  **(ng/mL)** | 9.1 (4.2) | 10.0 (4.4) | 11.7 (5.6) | 12.4 (7.7) |
| **MMP9***  **(ng/mL)** | 163.4 (100.6) | 230.3 (217.3) | 243.3 (256.4) | 253.4 (201.7) |
| **CXCL5**  **(ng/mL)** | 4.6 (5.8) | 4.3 (5.9) | 3.2 (3.4) | 3.2 (2.9) |
| **CXCL9****  **(pg/mL)** | 689.5 (331.9) | 1025.0 (1105.0) | 1735.0 (3680.3) | 1484.0 (1303.0) |
| **ICAM-1****  **(ng/mL)** | 103.3 (34.1) | 119.1 (45.4) | 142.7 (71.5) | 129.3 (50.7) |
| **VCAM-1****  **(ng/mL)** | 525.1 (180.7) | 542.9 (164.1) | 600.3 (181.3) | 601.0 (197.8) |

*p value <0.05 **p value <0.01 for trend test across strata, Jonckheere’s trend test.

Stratum 1 = Lifetime non-smokers

Stratum 2 = Smokers without airflow obstruction. FEV_1_/FVC >0.70

Stratum 3 = Mild to moderate airflow obstruction. FEV_1_/FVC <0.70, FEV_1_ >50%predicted

Stratum 4 = Severe to very severe airflow obstruction. FEV_1_/FVC <0.70, FEV_1_ <50%predicted

CRP = C-reactive protein (CRP). IL-6 = Interleukin-6. TNF-α = Tumor necrosis factor-alpha. MMP = Matrix metalloproteinase. CXCL = C-X-C Motif Chemokine Ligand. ICAM = Intercellular adhesion molecule. VCAM = Vascular cell adhesion molecule.

**Additional file 1: Table S2: Correlation Between Emphysema and Coronary Artery Calcification**

|  | **Coronary Artery Calcification**^‡^  (r, p value) | **Emphysema**^*^  (r, p value) |
| --- | --- | --- |
| **CRP**  **(ug/mL)** | 0.097, 0.096 | 0.131, 0.023 |
| **Fibrinogen**  **(mg/mL)** | 0.136, 0.019 | 0.151, 0.009 |
| **IL-6**  **(pg/mL)** | 0.095, 0.101 | 0.076, 0.187 |
| **TNF-α**  **(pg/mL)** | 0.016, 0.780 | 0.056, 0.335 |
| **MMP3**  **(ng/mL)** | 0.257, <0.001 | 0.179, 0.002 |
| **MMP9**  **(ng/mL)** | 0.034, 0.555 | 0.041, 0.483 |
| **CXCL5**  **(ng/mL)** | -0.262, <0.001 | -0.202, <0.001 |
| **CXCL9**  **(pg/mL)** | 0.331, <0.001 | 0.307, <0.001 |
| **ICAM-1**  **(ng/mL)** | 0.161, 0.005 | 0.079, 0.174 |
| **VCAM-1**  **(ng/mL)** | 0.249, <0.001 | 0.158, 0.006 |

r = Pearson’s correlation coefficient.

* Quantitative Emphysema on computed tomography, defined by percentage low attenuation areas <-950HU on density mask analysis. Log transformed

^‡^Visual coronary artery calcium scores assessed using the Weston score, log transformed.

CRP = C-reactive protein (CRP). IL-6 = Interleukin-6. TNF-α = Tumor necrosis factor-alpha. MMP = Matrix metalloproteinase. CXCL = C-X-C Motif Chemokine Ligand. ICAM = Intercellular adhesion molecule. VCAM = Vascular cell adhesion molecule.

**Additional file 1 Table S3: Comparison of Plasma Biomarkers by Visual COPD Subtype**

| **Plasma mediators‡** | **Substantial Centrilobular Emphysema** | | **Substantial Paraseptal Emphysema** | | **Definite Airway Wall Thickening** | |
| --- | --- | --- | --- | --- | --- | --- |
|  | Yes (n= 110) | No (n= 190) | Yes (n=40) | No (n= 260) | Yes (n=51) | No (n=249) |
| **CRP**  **(ug/mL)** | 5.7 (8.1) | 4.0 (6.3) | 3.7 (3.0) | 4.8 (7.5) | 6.1 (10.2) | 4.4 (6.2) |
| **Fibrinogen**  **(mg/mL)** | 5.1 (1.3)** | 4.7 (1.1) | 4.9 (1.1) | 4.8 (1.2) | 5.1 (1.2)* | 4.7 (1.1) |
| **IL-6**  **(pg/mL)** | 20.5 (138.2) | 4.7 (8.6) | 3.6 (1.0) | 11.4 (90.3) | 4.7 (4.6) | 11.5 (92.3) |
| **TNF-α**  **(pg/mL)** | 16.8 (74.4) | 9.3 (2.2) | 10.9 (5.4) | 12.2 (48.4) | 9.4 (2.2) | 12.6 (49.5) |
| **MMP3**  **(ng/mL)** | 11.9 (7.0)* | 10.1 (4.9) | 11.0 (5.7) | 10.8 (5.8) | 12.3 (8.1) | 10.5 (5.2) |
| **MMP9**  **(ng/mL)** | 255.7 (208.9)* | 203.4 (199.7) | 190.0 (158.6) | 227.6 (210.3) | 292.5 (268.5)* | 208.2 (186.0) |
| **CXCL5**  **(ng/mL)** | 3.0 (3.1)* | 4.3 (5.3) | 3.6 (5.2) | 3.8 (4.6) | 3.3 (3.4) | 3.9 (4.9) |
| **CXCL9**  **(pg/mL)** | 1648.8 (2862.7)* | 992.9 (1278.9) | 1457.9 (2237.4) | 1198.8 (1998.9) | 1226.3 (822.2) | 1234.8 (2198.4) |
| **ICAM-1**  **(ng/mL)** | 135.9 (69.0)** | 116.5 (41.4) | 132.6 (48.9) | 122.2 (54.6) | 131.6 (46.3) | 121.9 (55.3) |
| **VCAM-1**  **(ng/mL)** | 599.2 (181.8)* | 548.9 (182.6) | 586.9 (171.1) | 564.3 (185.6) | 562.7 (149.0) | 568.3 (190.2) |

*p<0.05

**p<0.01

‡All plasma mediators were log transformed prior to analysis.

CRP = C-reactive protein (CRP). IL-6 = Interleukin-6. TNF-α = Tumor necrosis factor-alpha. MMP = Matrix metalloproteinase. CXCL = C-X-C Motif Chemokine Ligand. ICAM = Intercellular adhesion molecule. VCAM = Vascular cell adhesion molecule.

**Figure Legend**

**Additional file 1: Figure S1: Mediation analysis model.**


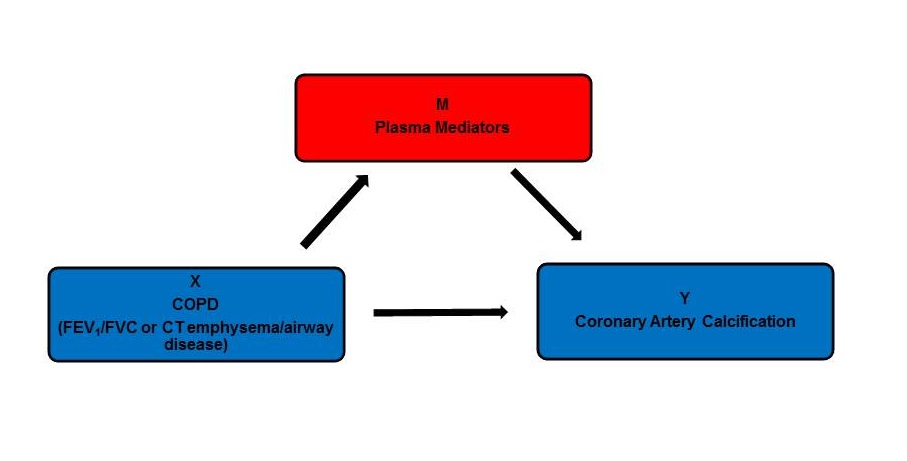


COPD = Chronic obstructive pulmonary disease. CT = Computed tomography. FEV_1_ = Forced expiratory volume in the first second. FVC = Forced vital capacity.

1. Sieren JP, Newell JD, Jr., Barr RG, et al. SPIROMICS Protocol for Multicenter Quantitative Computed Tomography to Phenotype the Lungs. *Am J Respir Crit Care Med* 2016; **194**(7): 794-806.

2. Hoffman EA, Simon BA, McLennan G. State of the Art. A structural and functional assessment of the lung via multidetector-row computed tomography: phenotyping chronic obstructive pulmonary disease. *Proc Am Thorac Soc* 2006; **3**(6): 519-32.

3. Grydeland TB, Dirksen A, Coxson HO, et al. Quantitative computed tomography: emphysema and airway wall thickness by sex, age and smoking. *Eur Respir J* 2009; **34**(4): 858-65.

4. Patel BD, Coxson HO, Pillai SG, et al. Airway wall thickening and emphysema show independent familial aggregation in chronic obstructive pulmonary disease. *Am J Respir Crit Care Med* 2008; **178**(5): 500-5.

5. Tschirren J, Hoffman EA, McLennan G, Sonka M. Intrathoracic airway trees: segmentation and airway morphology analysis from low-dose CT scans. *IEEE Trans Med Imaging* 2005; **24**(12): 1529-39.

6. Reinhardt JM, D'Souza ND, Hoffman EA. Accurate measurement of intrathoracic airways. *IEEE Trans Med Imaging* 1997; **16**(6): 820-7.
